# Enhanced nuclear fusion in the sub-keV energy regime

**DOI:** 10.1038/s41467-026-74421-1

**Published:** 2026-07-18

**Authors:** Micah E. Karahadian, Matthew Colborne, Arun Persaud, Thomas Schenkel, Jeremy N. Munday

**Affiliations:** 1 Department of Electrical and Computer Engineering, University of California, Davis, One Shields Avenue, Davis, CA USA; 2 Accelerator Technology and Applied Physics Division, Lawrence Berkeley National Laboratory, 1 Cyclotron Road, Berkeley, CA USA

**Keywords:** Materials for energy and catalysis, Experimental nuclear physics

## Abstract

Nuclear fusion requires overcoming or traversing a repulsive Coulomb barrier of hundreds of kiloelectronvolts, rendering the probability of fusion at sub-keV energies vanishingly small. Yet in condensed matter, the electronic and structural environment of reacting nuclei can profoundly alter fusion rates. Here we demonstrate that deuterium–deuterium fusion within metallic foils exhibits a pronounced reaction yield plateau (i.e., a finite, non-vanishing yield floor) below 2 keV—in stark contrast to the expected exponential suppression at low energy. At the lowest energies measured, this corresponds to fusion yields enhanced by more than 10¹⁸ relative to bare-nucleus (unscreened) expectations. Using a dual-chamber platform that combines electrochemical deuterium loading with low-energy ion-beam bombardment, we observe this behavior in both palladium and titanium hydrides. These results reveal a previously unrecognized regime of low-energy nuclear reactions in solids, demonstrating that materials degrees of freedom can fundamentally renormalize tunneling probabilities and fusion cross-sections.

## Abstract (web summary)

Fusion at very low energies is normally strongly suppressed. Here, authors show that deuterium fusion in palladium and titanium hydrides reaches a finite low-energy yield floor, revealing that solid materials can strongly reshape the nuclear reaction environment and alter nuclear reaction rates.

## Introduction

Nuclear fusion is the energy source of stars and a long-standing aspiration for terrestrial power generation. For light-nuclei fusion, Coulomb repulsion imposes a 100 s of keV-scale barrier. Quantum mechanical tunneling allows for ions to fuse without the need to overcome this barrier; however, reaction rates fall exponentially with decreasing ion energy or temperature^1^. Progress toward reliable control of fusion plasmas for power production has accelerated in recent years with a series of promising confinement approaches^2–5^; though, many challenges remain^6–8^. In parallel, there is renewed interest in probing fusion in solids, where mechanisms exist to enhance the fusion rates^9–12^.

Although fusion rates in condensed-matter systems are not presently relevant for energy production, understanding how electronic structure, defect landscapes, and hydrogen transport modify tunneling probabilities is increasingly important for low-energy nuclear reaction physics. Materials-driven modulation of nuclear reaction environments could help constrain models of screening, guide the design of enabling components for future fusion devices, and clarify whether solid-state environments provide regimes of fusion behavior not accessible in plasmas.

Ion beam experiments with solid targets provide a controlled means to study fusion at low reaction energies, where the electronic structure of the host material influences the reaction environment. Instead of colliding with bare nuclei, projectile ions interact with target nuclei embedded in materials, whose electron clouds can partially shield the nuclear charge. This phenomenon, referred to as electronic screening, lowers the effective Coulomb barrier, allowing nuclei to approach more closely and thereby enhance the tunneling probability compared with unscreened reactions^13,14^. Electron screening is quantified through a screening potential *U*_*e*_—adapted to metal hydrides by Assenbaum, Langanke, and Rolfs (ALR) in the context of low-energy astrophysical fusion reaction, which acts as an effective upward shift of the reaction energy, *E*_*eff*_ = *E* + *U*_*e*_, reducing the Coulomb barrier penetrability at low energies^1,15^. Values of *U*_*e*_ vary across materials from a few tens to several hundred eV^16–19^.

Prior reports point to the potential for fusion rate modification at low energies. Chen et al. demonstrated that electrochemical loading of deuterium (D) into a Pd foil increases the fusion yield by 15–18% at center-of-mass (cm) energies down to *E*_*cm*_ = 15 keV^10^. This experiment showed that meV-scale electrochemistry can affect MeV-scale fusion rates through an increase in the deuterium density within the Pd lattice, but was unable to observe the effects of electron screening, which are proposed to occur at lower energies^15,20,21^. At low energies, the complex interplay between electron screening, deuterium density, and ion energy requires a different approach where sub-keV deuterium ions can be used to systematically interrogate fusion rates across dissimilar hydrides under varied deuterium loading conditions.

Despite decades of study, the behavior of nuclear fusion cross-sections at extremely low energies in condensed matter remains poorly constrained. In this regime, standard models treat electron screening as a small, energy-independent correction, implicitly assuming a homogeneous reaction environment. Whether materials structure, defect production, or hydrogen transport can qualitatively alter tunneling behavior at sub-keV energies remains an open question. Recent experimental studies of nuclear fusion have revealed unexpected physical behavior across a wide range of regimes, from suprathermal ion distributions in burning plasmas to fast-ion–mediated turbulence suppression in magnetically confined systems, as well as non-hydrodynamic effects in inertial fusion experiments^22–25^. These results highlight that fusion reactions remain sensitive to local environments and collective effects beyond conventional theoretical descriptions.

Here, we report direct measurements of deuterium–deuterium fusion in metallic hydrides at center-of-mass energies spanning 0.25–6.5 keV and observe the emergence of a pronounced fusion yield plateau (i.e., a finite yield floor) below ~2 keV, which cannot be captured by standard ALR screened tunneling models. Experiments were performed by combining very low-energy deuteron beams with electrochemical loading in palladium and titanium foils, and fusion yields were quantified using proton detection, with neutron measurements providing independent validation in the higher-energy regime. This behavior is reproducible across two distinct host materials.

The observation of a low-energy plateau represents a qualitative departure from the expected exponential suppression of fusion probability at decreasing energy^26^, indicating that the reaction environment in condensed matter cannot be treated as homogeneous in this regime. These results establish a previously unrecognized materials-governed domain of low-energy nuclear reactions and motivate new theoretical descriptions of tunneling in heterogeneous solid-state environments.

## Experimental set-up

**Fig. 1:**
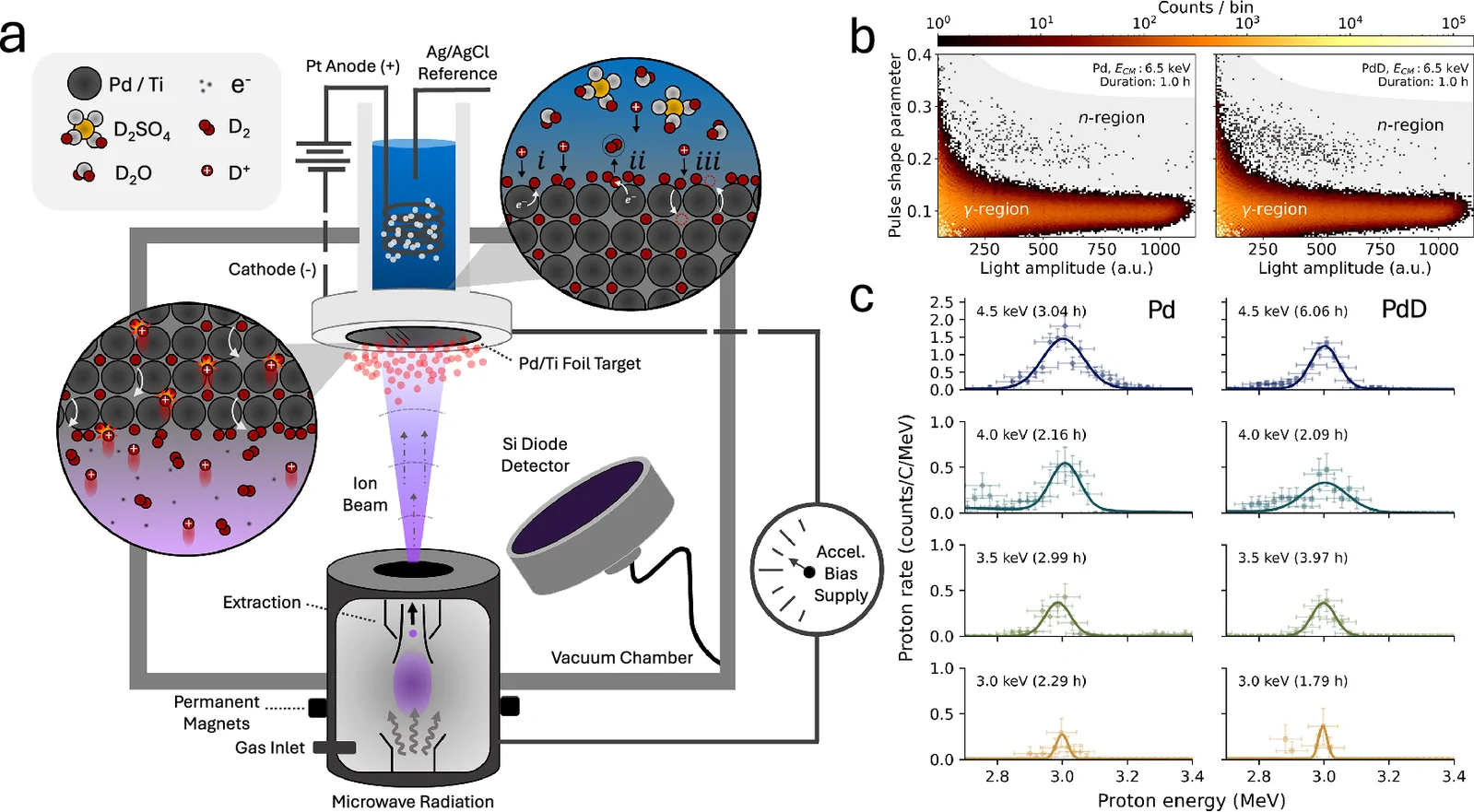
Experimental platform for probing sub-keV fusion in metal hydrides. **a** Schematic of the dual-chamber configuration, in which a Pd or Ti foil separates an electrochemical cell (top) from a high-vacuum ion-beam chamber (bottom), enabling simultaneous electrochemical deuterium loading and low-energy deuteron implantation (*E*_*cm*_ = 0.25–6.5 keV). On the electrolyte side, interfacial deuterium coverage and uptake are governed by the canonical (i) Volmer, (ii) Tafel/Heyrovský, and (iii) subsurface exchange^54^ (see Electrochemical Cell in Methods). Charged particles (notably 3.02 MeV protons) are recorded with a silicon diode; neutrons are detected with an organic liquid scintillator coupled to a photomultiplier (not shown). **b** Pulse-shape-discrimination (PSD) maps from the neutron detector at *E*_*cm*_ = 6.5 keV for beam-only loaded Pd (left) and electrochemically loaded PdD (right), showing clear separation between γ-ray and neutron events. The γ-ray events in the PSD map arise from ambient laboratory background (natural radioactivity and cosmic-ray secondary emission), not from D–D fusion products. Pulse-shape discrimination rejects these events with >98% efficiency; D–D fusion neutrons are selected in the upper (neutron) band. **c** Proton energy spectra measured with a silicon diode for Pd (beam-only) and PdD (beam plus electrochemical loading), exhibiting the characteristic ~3 MeV fusion proton peak across multiple energies. Together, these diagnostics enable independent, background-suppressed measurements of D–D fusion yields under controlled materials conditions.

Experiments were performed with palladium and titanium foils (250 µm thick) serving as membranes between an electrochemical cell and a high-vacuum ion-beam chamber (Fig. 1a). At the beam energies used here (*E*_*cm*_ = 0.25–6.5 keV), the D⁺ implantation depth ranges from ~4 nm at the lowest energies to ~70 nm at the highest, confining all beam-induced fusion reactions to a thin near-surface layer well under 0.1 μm in depth^27^ and separated from the electrochemical cell by the full 250 μm foil thickness. Palladium, with its high deuterium mobility and near-unity terminal solubility (D/Pd ≤ 1)^28^, provides a benchmark system where strong electron screening has long been reported^29,30^. Titanium, by contrast, despite a higher solubility limit (D/Ti ≈ 2), exhibits lower diffusivity and is widely regarded as weakly screening^16,17,19,31^.

Electrochemical loading of deuterium was carried out in a heavy-water electrolyte (6.0 M D_2_SO_4_ in D_2_O) under galvanostatic control, driving deuterons from solution into the metal lattice. To facilitate efficient chemisorption in Ti, the electrolyte-facing surface was coated with a thin Pd layer. A pressure transducer and residual-gas analyzer (RGA) tracked permeating hydrogen in real time, where pressure increases linearly with applied current density (Supplementary Fig. 1). On the vacuum side, the same foil was simultaneously bombarded with a monoenergetic deuteron beam produced by a microwave-driven ion gun, tunable between *E*_*cm*_ = 0.25 keV and 6.5 keV. Comparative chemical, structural, and morphological analyses of pristine and irradiated targets were carried out and are consistent with the expected degradation associated with ion beam bombardment (Supplementary Fig. 2). Nuclear fusion products were monitored in two of the D-D reaction channels: 2.45 MeV neutrons with a liquid scintillator coupled to pulse-shape-discriminating electronics (Figs. 1b) and 3.02 MeV protons with a passivated implanted planar silicon diode (Fig. 1c). Detector calibrations, beam flux normalization, and detailed descriptions of the electrochemical and ion-beam systems are provided in Methods. This configuration allows direct comparison between beam-only and beam-plus-electrochemical loading conditions, enabling systematic measurements of screening potentials across two canonical hydrides (PdD and TiD) in an unprobed energy regime where electron screening is expected to dominate fusion dynamics.

## Results

**Fig. 2:**
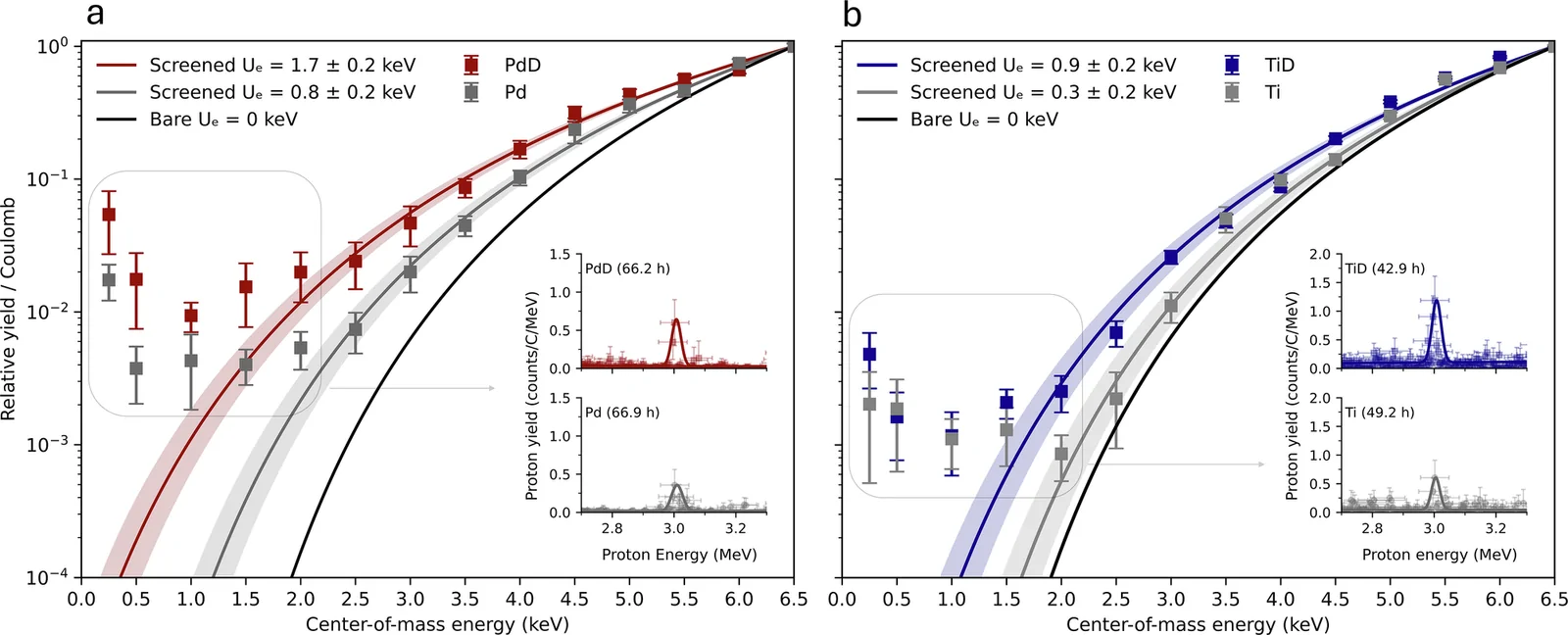
Emergence of a low-energy fusion yield plateau (i.e., a finite yield floor) in metal hydrides. **a** Relative yields for Pd (beam-only) and PdD (beam plus electrochemical loading), normalized to the highest-energy point (*E*_*cm*_ = 6.5 keV). Solid curves show the ALR screened thick-target yield with constant effective screening potential *U*_*e*_ fitted to data in the *E*_*cm*_ = 2.5–6.5 keV range; the unscreened (“bare”) case is shown for reference (*U*_*e*_ = 0). Shaded bands represent the ±0.2 keV uncertainty in the fitted *Uₑ* propagated through the yield model. Curves are extrapolated to lower energies to illustrate the systematic departure in the plateau regime. While the screened model captures the data above ~2.5 keV, a pronounced yield plateau emerges at lower energies. **b** Corresponding relative yields for Ti and TiD, showing similar low-energy behavior despite distinct electronic and transport properties. The deviation from constant-screening models below ~2 keV indicates the onset of a distinct low-energy fusion regime. Insets: cumulative 3 MeV proton spectra integrated over the plateau energy range. Poisson counting uncertainties are propagated through the normalization, and solid lines are included as a guide to the eye.

The measured fusion yield separates into two qualitatively distinct regimes: a screening-dominated regime above ~2.0 keV and a low-energy plateau (i.e., a finite yield floor) regime below this threshold, where the yield is statistically constant within measurement uncertainty across consecutive energy points. The plateau onset is defined here as the energy below which the relative yield is no longer decreasing within the statistical measurement uncertainty—a model-independent criterion based on the consistency of the data from point-to-point rather than departure from any theoretical prediction. The inset boxes in Fig. 2a, b encompass the integrated proton spectra across the five lowest energy points (*E*_*cm*_ = 0.25, 0.5, 1.0, 1.5, and 2.0 keV). This finite yield floor indicates the emergence of additional effects that are not captured by homogeneous screened tunneling models in this reaction energy regime. Figure 2 shows the relative fusion yields for metal targets upon ion bombardment alone (Pd and Ti) and with electrochemical loading of deuterium (PdD and TiD) across both regimes. Solid curves represent the ALR screened thick-target yield in which electron screening is parameterized by a single effective energy shift *Uₑ* applied to the Gamow tunneling factor; *Uₑ* is extracted by fitting to the *E*_*cm*_ = 2.5–6.5 keV regime. The complete model, including the cross-section expression, thick target yield integral, and stopping power treatment, is given in Methods. In these experiments, the detected proton signals dropped to background levels during electrochemical loading in the absence of ion beams. Proton and neutron counts are scaled per Coulomb using Faraday-cup measured currents and are normalized to each material’s highest-energy reference point (*E*_*cm*_ = 6.5 keV) to achieve the relative yield. This procedure (details in Methods, Supplementary Fig. 3) enables direct comparisons between metals and their hydrides and consistent extraction of the effective screening potential *U*_*e*_ for each material.

Fitting the relative yields with ALR screened cross-section models^15^ (see Methods), we find that the effective screening potential in Pd increases from *U*_*e*_ = 0.8 ± 0.2 keV in the beam-loaded foil to 1.7 ± 0.2 keV with beam plus electrochemical loading. For Ti, the corresponding increase is from *U*_*e*_ = 0.3 ± 0.2 keV to 0.9 ± 0.2 keV. Thus, while electrochemical loading introduces a similar increment of approximately 0.6 to 0.9 keV to the apparent screening potential, the absolute magnitude remains much larger in the Pd system.

**Fig. 3:**
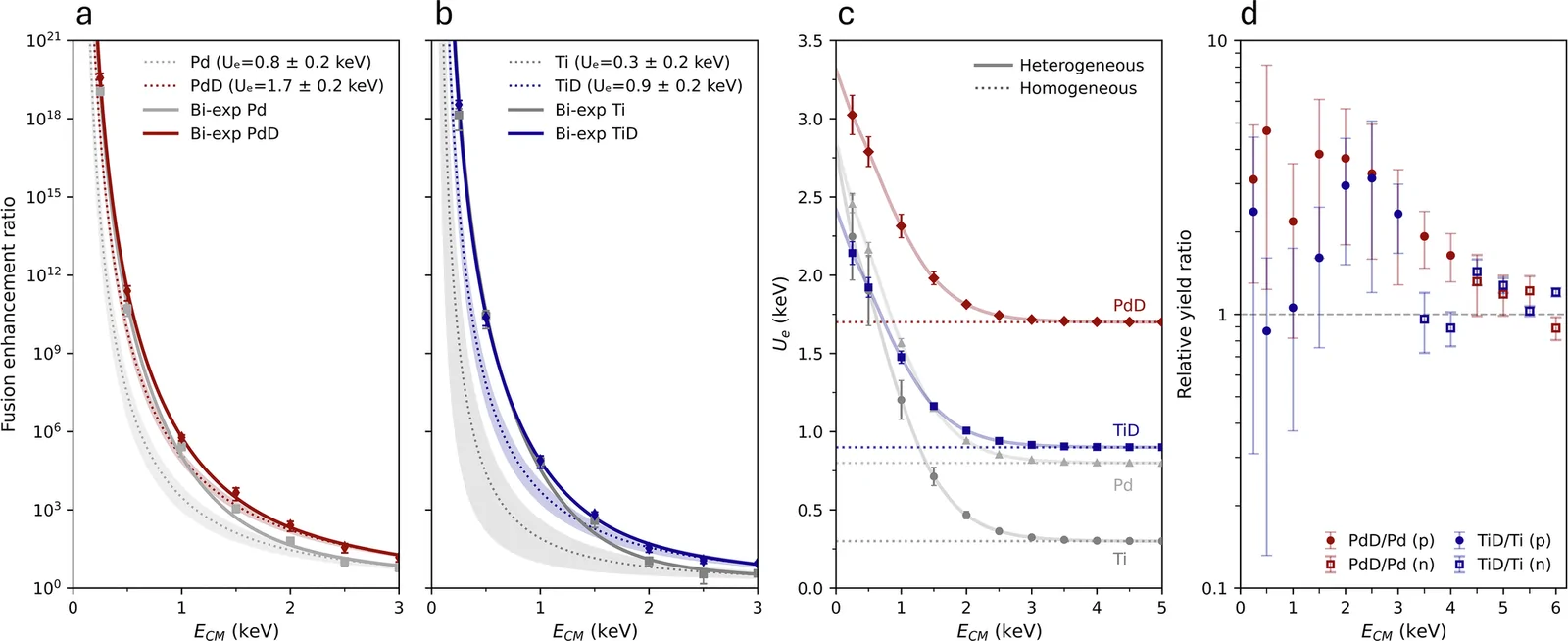
Breakdown of standard screening models, phenomenological description of low-energy enhancement. **a**, **b** Fusion enhancement factor relative to bare-nucleus expectations for **a** Pd and PdD and **b** Ti and TiD as a function of center-of-mass energy. Dotted curves show the behavior expected from constant homogeneous screening models using screening potentials extracted from the higher-energy data (*E*_*cm*_ = 2.5–6.5 keV); these models fail systematically below ~2.0 keV. Solid curves show the bi-exponential heterogeneous screening model that captures the curvature of the enhancement across the full energy range. Shaded bands reflect the ±0.2 keV uncertainty in *U*_*e*_ propagated through the enhancement factor. **c** Per-energy effective screening potential *U*_*e*_ extracted from the bi-exponential model fit to the measured enhancements in panels (**a**, **b**). Dotted lines show the high-energy homogeneous asymptotes while solid curves show the heterogeneous model fit (see Screening Enhancement and Model Fit in Methods). **d** Hydride-to-metal relative yield ratio for Pd and Ti across proton (p) and neutron (n) detection channels, showing consistent enhancement upon electrochemical loading. Error bars denote 1σ counting uncertainties propagated through the yield normalization.

We compare the fusion rate in the metal foils to the expected fusion rate of bare, unscreened nuclei^26^ by defining a fusion enhancement factor, which is the ratio of the measured screened yield to the bare (unscreened) yield at the same center-of-mass energy: ${{\mathscr{E}}}\left(E\right)={Y}_{{{\rm{scr}}}}\left(E\right)/{Y}_{{{\rm{bare}}}}\left(E\right)$. Figure 3 shows ${{\mathscr{E}}}\left(E\right)$ for (a) Pd and PdD and (b) Ti and TiD.

The dotted curves in Fig. 3a show the behavior expected from a parameter-free ALR screened tunneling model, constructed using screening potentials extracted from the higher-energy regime (2.5–6.5 keV). While this description captures the data at higher energies, it fails systematically below ~2 keV for both materials studied. To parameterize this deviation without assuming a specific microscopic mechanism, we introduce a phenomenological model (solid curves) consisting of the sum of two exponential functions that depend on energy—a ‘bi-exponential’ form capturing a tunneling-dominated process with two characteristic energy scales across the full energy range. These components may correspond to reaction environments with distinct local screening strengths, such as near-surface defect-trapped deuterium and deeper bulk-dissolved deuterium. The complete mathematical form and fitting procedure are given in Methods. This representation is not intended as a unique physical model, but rather as a compact description of heterogeneous reaction environments such as near-surface defect-rich regions and more bulk-like lattice environments that become increasingly relevant at low energies as the beam probes the surface of the metallic target.

At the lowest reaction energies in our experiments, the fusion enhancement factor approaches or exceeds 10^18^ for all materials considered (Pd, PdD, Ti, and TiD). Figure 3c shows the per-energy effective screening potential extracted from the bi-exponential model at each beam energy. At high energies (*E*_*cm*_ = 2.5–6.5 keV), the effective screening potential converges to the bulk homogeneous values of 0.8 keV (Pd) and 0.3 keV (Ti), consistent with previously reported values for these systems^16–19,31^. With decreasing beam energy, *U*_*e*_ rises continuously, reaching approximately 3.0 keV for PdD and 2.2 keV for TiD at *E*_*cm*_ = 0.25 keV—the values required within the ALR framework to reproduce the observed enhancement of 10^18^. This energy dependence is consistent with depth-dependent environmental sampling: lower-energy ions stop progressively closer to the surface (~ 4 nm at *E*_*cm*_ = 0.25 keV), where defect density and non-equilibrium deuterium occupancy are highest. The smooth, monotonic rise of *U*_*e*_ from the bulk constant values at high energy to larger values at low energy therefore encodes the evolving mixture of local reaction environments sampled by the beam, rather than a single homogeneous screening condition.

Figure 3d shows the hydride-to-metal relative yield ratio, ${Y}_{{{\rm{hydride}}}}/{Y}_{{{\rm{metal}}}}$, for both material systems, showing that electrochemical loading increases the yield in both metal hosts and trends toward unity at higher energies, as expected. Across the measured ranges, the relative yield ratio for both Pd and Ti is broadly similar and overlaps within uncertainties. Proton- and neutron-based ratios show similar enhancements, indicating that the loading effect is consistent across diagnostics.

### Experimental controls and systematic checks

Several experimental controls and cross-checks confirm that the observed low-energy finite yield floor arises from nuclear fusion events rather than experimental artifacts (further details can be found in the Methods). Fusion signals are detected independently in both proton and neutron reaction channels, with consistent energy dependence in the overlapping measurement regime, as expected from the nearly equal branching ratio of the D–D fusion reactions. No excess proton or neutron counts at the energy channels detected in these experiments are observed during electrochemical loading in the absence of an ion beam, indicating that electrochemical processes alone do not generate detectable fusion-like signals above the background in these experiments. Background measurements accumulated over more than 350 h remain stable over time. The ion energy spread (≈ 5%) and beam-current normalization were independently characterized and are insufficient to account for the emergence of a yield plateau at low energies. Finally, identical detector geometries, acquisition settings, and analysis procedures were used across all energies and materials, ensuring that the observed behavior does not arise from changes in experimental configuration.

## Discussion

A central experimental finding of this work is the emergence of a pronounced plateau—corresponding to a finite yield floor—in the deuterium–deuterium fusion yield below ~2 keV in metallic hydrides, in stark contrast to the expected exponential suppression at low energies. This behavior is observed reproducibly in both Pd and Ti hosts. Standard screened cross-section models with constant screening potentials fail to capture this low-energy behavior across all materials studied.

Several mechanisms could plausibly contribute to the observed plateau behavior. Electrochemical loading introduces deuterium into lattice interstitials and can modify the local electronic environment surrounding reacting nuclei^32–34^. In parallel, low-energy ion implantation generates near-surface defects and vacancies^35^ that can trap deuterium and locally increase reacting pair density^36–40^. Such defect-mediated near-surface environments can create spatially heterogeneous tunneling conditions within the implantation depth, particularly under simultaneous ion irradiation and electrochemical loading. Notably, pre-existing grain-boundary defects and displaced hydrogen in Pd can amplify electron screening potentials by up to an order of magnitude above the adiabatic limit, with the enhancement directly correlated to hydrogen occupancy at non-equilibrium lattice sites^41^. This mechanism is consistent with the near-surface defect landscape created by our ion beam, where displacement cascades and vacancy formation are unavoidable at sub-keV energies. However, the appearance of a plateau for both Pd and Ti—materials with substantially different defect production thresholds and hydrogen transport coefficients—near the same ion beam energy suggests a general, possibly multi-mechanism origin that includes but is not limited to defect-enhanced screening. These effects may coexist with depth-dependent screening environments, as ions of different energies probe distinct regions of the near-surface lattice^27^. In addition, it has been proposed that low-energy nuclear resonances could contribute to anomalous fusion behavior in solids^42,43^. Distinguishing among these possibilities will require further experiments, including searches for correlated internal e⁺e⁻ pair emission^44^ for a series of materials, loading and excitation conditions.

At higher energies (*E*_*cm*_ = 2.5 keV), our experimentally determined relative thick-target yields across different materials are well described by screened-tunneling models with a single effective screening potential^15–21^ and are affected by the evolving electronic structure driven by hydrogen loading^32–34^. However, the breakdown of the single-parameter screened-tunneling models below *E*_*cm*_ = 2 keV indicates that the fusion reaction environment in condensed matter cannot be treated as homogeneous; instead, the data imply that local variations in electronic structure, defect density, and deuterium distribution strongly modulate tunneling probabilities at sub-keV energies.

Differences between Pd and Ti may provide useful context for interpreting the observed screening behavior for *E*_*cm*_ ≥ 2.5 keV. Pd remains metallic upon hydrogen uptake, retaining a substantial density of states at the Fermi level, whereas Ti and its hydrides are less conductive with fewer itinerant charge carriers^32–34,45–47^. These electronic differences are consistent with the larger effective screening potentials extracted for Pd-based systems relative to Ti-based systems at higher reaction energies, even though both materials exhibit qualitatively similar low-energy plateau behavior.

While an electron screening model has been employed to describe fusion rates in metal targets, other effects could also be at play. Deuterium diffuses more rapidly in Pd than Ti^48,49^, allowing it to move more freely and replace vacancies or depleted regions within the foil more readily than Ti. Under ion beam irradiation, the near-surface deuterium concentration reflects a balance between beam implantation, thermally and ion-stimulated losses to vacuum, and diffusive replenishment from the bulk during electrochemical loading. Because Pd exhibits much higher hydrogen mobility than Ti, Pd can more readily sustain the near-surface population required for low-energy reactions under otherwise similar loss fluxes, whereas Ti could develop a relatively depleted layer when diffusion and de-trapping cannot keep pace. These properties may also play a role in the different fusion yields for these materials. The results of several effects may be lumped into one parameter, the effective screening potential *U*_e_, even though they are physically different mechanisms.

This work establishes a new experimental regime in which nuclear fusion at sub-keV energies can be reproducibly controlled by the materials environment. By coupling electrochemical deuterium loading with low-energy ion beam excitation, we uncover a plateau in D–D fusion yield below 2 keV that defies the expected exponential suppression. By comparing relative thick-target yields from Pd and Ti foils with and without electrochemical loading, we extract screening potentials of 1.7 ± 0.2 keV for PdD and 0.9 ± 0.2 keV for TiD—substantially higher than their non-electrochemically loaded counterparts, while suggesting that the local electronic and defect structures of metals actively shape tunneling probabilities.

The fusion enhancements observed here, reaching over 10^18^ times the bare-nucleus rate at an ion beam energy of 0.25 keV, demonstrate that nuclear reaction probabilities can be strongly modulated by the condensed-matter environment. The broadly similar behavior observed for Pd and Ti, despite their distinct electronic structures and lattice properties, suggests that this effect is general across metal hydride systems and provides a framework for systematic exploration across a wider class of materials. A key open question is the microscopic origin of the increased effective screening potentials at low energies. Depth-resolved hydrogen profiling (e.g., Secondary Ion Mass Spectrometry, Nuclear Reaction Analysis or Elastic Recoil Detection Analysis) and open-volume defect spectroscopy (Positron Annihilation Spectroscopy) under beam conditions would directly test the heterogeneous trapping hypothesis and constrain the relative contributions of near-surface defect populations and bulk deuterium occupancy to the measured enhancement.

These findings establish a reproducible experimental platform for fundamental studies of nuclear processes in solids. They provide a basis for mapping the dependence of reaction rates on composition, defect density and hydrogen transport properties, opening a systematic experimental space for exploring how material properties modulate nuclear processes. Future experiments can help delineate microscopic mechanisms with experiments that can include tests with ion beams other than deuterium and other excitation sources, including photons, THz phonons, and plasmons. More broadly, these findings establish condensed matter as an active degree of freedom in low-energy nuclear reaction physics. By revealing a controllable, materials-governed fusion regime, this work motivates new theoretical descriptions of tunneling in heterogeneous environments and opens experimental access to reaction energies previously considered inaccessible.

## Methods

### Materials

High-purity palladium and titanium foils (Goodfellow or Sigma-Aldrich; equivalent stated purities) were used as membrane substrates separating the electrolyte from the high-vacuum ion-beam chamber. Palladium foils (thickness 250 µm, ≥ 99.95% purity) and titanium foils (thickness 250 µm, ≥ 98.5% purity) were cut to the required aperture size and handled exclusively with non-marring, solvent-rinsed tools to minimize adventitious contamination. To promote efficient deuterium uptake on titanium, the electrolyte-facing side of each Ti foil was sputter-coated (Lesker Labline Sputter System) with a ∼250 nm palladium layer in a Class-100 workspace. The partially filled Pd 5*s*/4*d* orbital is known to support strong back-bonding interactions with hydrogen and serves as a catalytic overlayer for chemisorption and exchange^50,51^_._

Prior to assembly, all foils underwent a standardized cleaning sequence and thermal conditioning (details of annealing parameters provided in Cathode Preparation). Briefly, substrates were sonicated sequentially in acetone, ethanol, and 18 MΩ · cm deionized water, then dried under filtered nitrogen. Materials from both vendors were used interchangeably at the same nominal purity, and samples were tracked throughout to enable cross-checks of reproducibility.

### Cathode preparation

Cathodes were prepared as 2.4 cm-diameter disks using a precision punch. After deposition, samples were stored in clean containers and transferred directly to the vacuum flange for sealing to limit ambient reoxidation prior to operation. All cathode membranes (Pd and Ti) were prepared using an identical unload/recondition protocol to standardize microstructure across experiments. For each unloading cycle, foils were annealed at 400 °C for 3 h in high vacuum ( ≤ 1 × 10^−6^ Torr, 1.3 × 10^−6^ mbar) to relieve residual stress, heal sputter-induced damage, and desorb weakly bound surface species. Foil samples were allowed to cool under vacuum to near ambient in inert gas before exposure to air.

To restore a uniform vacuum-facing surface and to minimize run-to-run variations in crystallinity, a 200 nm metallic overlayer was sputter-deposited on the vacuum side after each anneal: Pd was applied to Pd foils and Ti to Ti foils (DC magnetron, Ar working pressure 10 mTorr (0.013 mbar), elevated substrate temperature). This reconditioning step served two purposes: (i) reset the near-surface grain structure prior to beam exposure, and (ii) partially replenish material eroded by prior beam sputtering. We note that this replenishment is not a strict 1:1 compensation for sputter loss; thickness and composition were tracked by deposition time-rate calibration, whereas net erosion during experiments was estimated separately from beam fluence and sputter yields and was not forced to match the added film.

### Vacuum system and diagnostics

Experiments were performed in a 6” (15.24 cm) stainless-steel high-vacuum chamber evacuated by a Pfeiffer HiCube 80 Eco turbomolecular pump with membrane backing (nominal 200 L min⁻¹). Base ( ≤ 10^−6^ Torr, 1.3 × 10⁻^6^ mbar) and operating (2.5 to 3 × 10^−4^ Torr, 4 × 10^−3^ mbar) pressures were monitored with an MKS 390411-0-YE-T hot-cathode ion gauge; residual gas composition was tracked with a Pfeiffer Prisma QME-200 quadrupole residual-gas analyzer (RGA).

### Ion source and beam delivery

Deuterium ions were produced with a filament-less microwave source (Tectra IonEtch), where microwave electron cyclotron resonance coupling with a quadrupole magnetic field (ECR confinement) sustained a stable discharge. Ions were extracted through three-grid, single-aperture optics that permit low-energy operation, down to ~25 eV without plasma collapse. For fusion rate measurements, the ion source delivered 0.5–5 kV beams through a 3 mm extraction aperture positioned ~4 cm from the target, forming a ~ 1 cm^2^ spot on the membrane. Ions could be accelerated to an energy up to 15 keV using a negative bias on metal targets. The ion beam current was typically ~150 µA and consisted predominantly of D^+^ (~85%) with the remainder as D_2_^+^ (~15%), consistent with microwave ECR sources where energetic, well-confined electrons rapidly dissociate molecular ions before extraction^52,53^. The beam current was monitored with a Faraday cup (Supplementary Fig. 3). Ion currents could also be monitored through measurements of the effective current from foil targets, where the contribution of secondary electrons was corrected for with Faraday cup measurements. The base pressure was ≤1 × 10^−6^ Torr (1.3 × 10^−6^ mbar); during operation, D₂ ( ≥ 99.98%) was metered with a needle valve to maintain 2.5–3 × 10^−4^ Torr (4 × 10^−3^ mbar), limiting scattering and charge-exchange of ions while preserving source stability and beam-on-target uniformity.

With a ~ 85/15 D⁺/D₂⁺ mixture from the microwave drive ion source, the deuteron flux at the target is

1 $${F}_{D}=\frac{I}{e}\frac{\left(1\times 0.85+2\times 0.15\right)}{A}\approx 1.1\times {10}^{15}D \cdot {{{\rm{cm}}}}^{-2}{{{\rm{s}}}}^{-1}\left(\approx 1.8{{\rm{nmol}}} \cdot {{{\rm{cm}}}}^{-2}{{{\rm{s}}}}^{-1}\right)$$

Where *I* is the beam current, *e* is the electron charge, and *A* is the irradiated area. Taking a representative implantation/mixing depth of *d*_*mix*_ = 200 nm (1–15 keV D ranges), the time to reach terminal solid solubility is set by the required deuteron inventory per unit area,

2 $${N}_{{req}}=x{n}_{m}{d}_{{mix}}$$

where *x* is D per metal atom at saturation (Pd: *x* = 1, Ti: *x* = 2), ${n}_{m}$ is the metal atomic number density (${n}_{{Pd}}=6.8\times {10}^{22}{{\rm{c}}}{{{\rm{m}}}}^{-3}$, ${n}_{{Ti}}=5.7\times {10}^{22}{{\rm{c}}}{{{\rm{m}}}}^{-3}$). Solving for the time needed to load to each metal's terminal solubility we have,

3 $${t}_{{load}}=\frac{{N}_{{req}}}{{F}_{D}}$$

For D/Pd = 1, ${t}_{{load}}\approx 21\min$ and for D/Ti = 2, ${t}_{{load}}\approx 35\min$. For a deeper 1 µm region, the corresponding times scale linearly ( ≈ 105 min for Pd to D/Pd = 1; ≈ 2.9 h for Ti to D/Ti = 2 at 150 µA). Because data-acquisition windows were several hours, the near-surface region operated in a dynamic steady state during measurements, with implantation balanced by diffusion and surface loss. Electrochemical loading was optionally applied in parallel to raise the near-surface chemical potential.

### Electrochemical cell

Electrochemical loading used a three-electrode geometry in 6 M D_2_SO_4_ prepared in D_2_O, with the membrane (Pd or Pd-coated Ti; electrolyte-facing side) serving as the working cathode, a platinum counter electrode, and an Ag/AgCl reference electrode. A potentiostat/galvanostat operated in galvanostatic mode at 300–700 mA cm^−2^ to drive D^+^ reduction and insertion using a CHI660D CH Instruments Electrochemical Workstation. When knowledge of exact electrochemical current was not needed, a DC power supply was used at 5 V in constant voltage mode. At the electrolyte–metal interface, deuterium enters the cathode through the standard sequence of surface steps^54^. First, the Volmer step produces adsorbed deuterium on the metal surface (charge transfer and adsorption).

4 $${{{\rm{D}}}}^{+}+{{{\rm{e}}}}^{-}\rightleftharpoons {{{\rm{D}}}}_{{{\rm{ads}}}}$$

Adsorbed deuterium is then removed either by the Tafel step (two adsorbates recombine to form D_2_) or by the Heyrovský step (an adsorbate combines with a solution ion during charge transfer to form D_2_).

5 $${{{\rm{D}}}}_{{{\rm{ads}}}}+{{{\rm{D}}}}_{{{\rm{ads}}}}\rightleftharpoons {{{\rm{D}}}}_{2\left({{\rm{g}}}\right)}$$

6 $${{{\rm{D}}}}_{{{\rm{ads}}}}+{{{\rm{D}}}}^{+}+{{{\rm{e}}}}^{-}\rightleftharpoons {{{\rm{D}}}}_{2\left({{\rm{g}}}\right)}$$

In parallel, adsorbed deuterium exchanges with subsurface sites (D_ads_ ⇌ D_abs_), providing the entry pathway into the bulk lattice. The balance among these steps sets the surface coverage of deuterium and, in turn, the net flux into the metal.

We deposit a thin (~250 nm) palladium film on the electrolyte-facing side of the titanium to ensure efficient loading. Titanium rapidly forms a stable oxide in aqueous media, which hinders the creation of surface deuterium and raises the potential required for interfacial charge transfer. Palladium, in contrast, readily forms and maintains a high coverage of adsorbed deuterium under the same conditions and catalyzes the Volmer and Heyrovský reactions^50,51^, lowering the barrier for deuterium to reach and populate the surface. Once formed on Pd, deuterium transfers across the Pd/Ti interface and enters the titanium lattice via the adsorbed–absorbed exchange (D_ads_ ⇌ D_abs_). The Pd layer is kept thin so that it governs only the interfacial chemistry and does not alter the bulk properties of the Ti target. The measured permeation flux scales linearly with the applied current density (Supplementary Fig. 1), confirming controlled, steady-state delivery of deuterium to the target. Teflon cell hardware and wetted materials were selected to be acid inert to minimize H/D back-exchange.

### Detector suite and electronics

Neutrons were recorded with a 5-inch EJ-309 liquid scintillator coupled to a Hamamatsu H6527 photomultiplier (bias 1600 V). Pulse-shape discrimination (charge-integration windowing) provided > 98% *n*/γ separation at operating thresholds. Charged particles were measured using a Canberra TCAM-450AM passivated implanted planar silicon diode that was reverse-biased at 60 V. To suppress spurious counts from low-energy secondary electrons emitted when D^+^ struck the membrane, the diode active area was covered with aluminum foil; the thickness was set to ~10 µm to balance proton transmission with secondary-electron rejection. Under these conditions, only protons penetrate to the active volume; tritons, ^3^He, and elastically scattered deuterons are range-limited and do not contribute. Signals from the diode channel are routed through low-noise preamplification (CAEN A1423B) to the digitizer (CAEN DT5730S) for event-mode acquisition; PSD and energy spectra were computed offline via CAEN DPP-PSD firmware. Detector distances, solid angles, thresholds, and live-time corrections were kept constant across runs to enable direct comparison of relative yields.

Energy loss and peak broadening of 3.02 MeV fusion protons traversing the detector geometry were estimated using SRIM Monte Carlo simulations^27^. For each beam energy, protons were propagated through the energy-dependent D^+^ implantation depth in each hydride target (4.4 nm at *E*_*cm*_ = 0.25 keV to 67.5 nm at *E*_*cm*_ = 6.5 keV), followed by the 10 μm Al entrance foil and 1.5 μm Si dead layer. The simulated FWHM of the transmitted proton energy distribution increases from ~35 keV at the lowest beam energies to ~39 keV at the highest, with the dominant contribution arising from the fixed Al absorber geometry. The in-target hydride contribution varies by less than 4 keV across the full explored range and does not introduce a systematic bias in the relative yield extraction. Detected proton spectra are energy-calibrated to the nominal 3.02 MeV Q-value, absorbing the fixed energy losses in the Al foil ( ~ 224 keV) and Si dead layer (~30 keV) into the calibration offset.

### Ion beam characterization

Beam current–voltage characteristics were measured with a Faraday cup placed at the target position and are shown in Supplementary Fig. 3. The ion source employs a three-electrode extraction system with constant extraction voltage maintained across all experiments. Two regimes are evident, separated by the onset of external bias above 5.0 kV. At or below this threshold, the beam operates with the internal gun bias only, and the measured current follows a saturating exponential form $I\propto 1-\exp (-V/{V}_{{ext}})$, reflecting the intrinsic beam transport characteristics through the fixed extraction optics. Above 5.0 kV, an external target bias was applied while maintaining constant extraction conditions. In this regime, the measured current increases as ${I\propto V}^{3/2}$, which we attribute to electrostatic lensing: as the target bias becomes more negative, the converging electric field focuses the beam to progressively tighter spot sizes at the Faraday cup aperture, or foil target surface. Since the total beam current remains constant but the spot size decreases with increasing bias, a larger fraction of the beam is captured by the fixed-diameter cup, resulting in higher measured current. This ${V}^{3/2}$ scaling is consistent with the beam’s transverse compression under increasingly strong focusing fields in this acceleration geometry. Error bars reflect repeat measurements and readout noise.

The ion energy spread was quantified with a retarding-potential (*V*_*R*_) analyzer formed by a metallic mesh mounted in front of the Faraday cup. A variable bias on the mesh creates an electrostatic barrier that transmits only ions with kinetic energy exceeding *qV*_*R*_. Sweeping *V*_*R*_ (2.5–4.2 kV) and recording the transmitted current gives the integrated energy distribution, and the differential spectrum is obtained. From these data (not shown), we infer an ion energy spread of ~5% at the operating energies.

### Beam-only runs

For D^+^ on stationary deuterium in the lattice, the center-of-mass energy, *E*_*cm*_, was derived from the acceleration potential, and operating points were chosen to avoid diode saturation from secondary-electron emission at high foil target voltages. In practice, secondary electrons limited reliable charged-particle spectroscopy above ~4 keV *E*_*cm*_ (material-dependent threshold; Pd vs Ti). Accordingly, neutron spectra were acquired over 4.5–7 keV *E*_*cm*_, while proton spectra were acquired over 4.5–0.25 keV *E*_*cm*_ where the neutron detector signal approached background levels in our setup. Because the two principal D–D branches (*p* + *t* and *n* + ^3^He) have approximately equal branching ratios, neutron and proton channels provide complementary information on the same fusion yield.

### Electrochemically loaded runs

For electrochemically loaded conditions, the same beam protocol and detector geometry were used. Loading proceeded under galvanostatic control until the cathode potential stabilized. Runs were rejected if chamber pressure, beam current, or cathode potential deviated beyond preset tolerances.

### Controls and backgrounds

Background measurements (beam off, no electrochemical loading) were interleaved with signal runs on the same geometry, yielding >350 h of accumulated background data. All spectra were corrected for live time; background spectra were scaled to the same live time and subtracted prior to yield extraction.

In the proton channel, the background rate is ~0.007 counts per hour (cph) in the integration window (2.7–3.3 MeV), measured over 352 h of accumulated beam-off, electrochemistry-off data. As shown in Supplementary Fig. 4, the rate is flat and stable across the full proton energy window, confirming no anomalous background structure near the 3.02 MeV fusion proton peak. The neutron channel background is substantially higher (~110 cph), a consequence of the EJ-309 scintillator’s larger active volume and sensitivity to cosmic-ray induced neutrons; statistically significant neutron detection is therefore limited to *E*_*cm*_ ≥ 3 keV where signal rates exceed the 3σ threshold within ~1 h runs.

Signal-to-background ratios for each beam energy and material condition are summarized in the supplementary table 1, where S:B is defined as the signal rate (cph) divided by the background rate (cph). Signal rates are computed as yield (counts/Coulomb) × beam current (A) × 3600 s/h using the measured beam current at each energy from the ion source *IV* characterization. S:B ratios range from >400:1 at *E*_*cm*_ = 3.0 keV to a minimum of 15:1 at *E*_*cm*_ = 0.5 keV for the Pd system and *E*_*cm*_ = 0.25 keV for Ti, confirming that all measurements are well above the detection threshold. At the lowest beam energies (*E*_*cm*_ ≤ 2.0 keV, denoted ^†^), run durations of 12–24 h per energy point compensate for the lower beam current and yield per Coulomb, maintaining robust signal-to-background ratios throughout the plateau regime. PdD and TiD consistently show higher S:B ratios than their beam-only counterparts at all energies, confirming that the electrochemical loading enhancement extends throughout the plateau region and is not an artifact of improved counting statistics alone.

### Yield extraction and screening metrics

Fusion products were counted by integrating calibrated pulse-height spectra over pre-defined regions of interest: (i) EJ-309 pulses passing neutron PSD cuts; (ii) proton energy windows in the Si diode centered on the 3.02 MeV line. Detector efficiencies included geometric solid angle and, for charged particles, secondary-electron suppression and entrance-foil transmission (∼10 µm Al). Backgrounds were subtracted before integration. All yields were reported per delivered Coulomb. For each run, we computed the total charge dose incident on the target using the Faraday cup-measured *IV* characteristic.

We convert the raw spectra into relative thick-target yields as a function of center-of-mass energy. The yields are normalized to data taken at the highest energies accessible in our setup, *E*_*cm*_ = 6.5 keV, where screening effects are small, and the measured cross-section converges to the unscreened (“bare”) value. This normalization removes dependence on absolute detector efficiency and beam current, enabling direct comparison between Pd, Ti, and their hydrides, allowing for accurate extraction of the effective screening potential, *U*_*e*_, for each system.

Screening enhancement factors were derived from relative thick-target yields. Specifically, counts accumulated within the designated “screening window” at low *E*_*cm*_ were normalized to the yield measured at higher energies outside this window (where screening effects are small) under identical geometry and detector settings. Uncertainties combined counting statistics (Poisson), background subtraction, and efficiency/solid-angle calibration errors in quadrature.

### Silicon diode energy calibration (α)

The energy calibration of the silicon diode detector (Canberra TCAM-450AM) was performed in vacuum using a ^241^Am α source (Eckert & Ziegler) mounted on a 25 mm-diameter, 0.5 mm-thick stainless disc. Three calibration anchors were used: the unattenuated *E*_*α*_ = 5.486 MeV line and the same line after transmission through 5 µm and 10 µm Al foils. Residual energies after each foil were computed from stopping-power tables and used as fixed references. Peak centroids (ADC) were obtained by Gaussian fits; a linear model was fitted by least squares to the three (ADC, E) pairs and then applied to all proton spectra. The fit residuals were ≪ 1–2% across the working range, and re-checks before/after long runs showed no measurable drift.

Uncertainty on *E*_*α*_ combines (in quadrature) the centroid-fit error, the foil-thickness tolerance propagated through *dE/dx*, and the diode bias stability; the source emission rate carries an expanded uncertainty of 3%, which was used when translating α rates to absolute detection efficiency (but not for the energy scale itself).

### Neutron PSD calibration and statistical selection (EJ-309)

Neutron/gamma separation for EJ-309 was acquired with CAEN CoMPASS software and subsequently analyzed with custom Python scripts. For each run, we formed the standard pulse-shape parameter PSP = (Q_long_−Q_short_)/Q_long_ vs light-output (ADC), where Q_short_ is the baseline-subtracted charge integrated over an early, short gate (from *t*_*0*_ to *t*_*0*_ + *T*_*short*_), capturing the prompt component of the pulse, and Q_long_ is the baseline-subtracted charge integrated over a longer gate (from *t*_*0*_ to *t*_*0*_ + *T*_*long*_, with *T*_*long*_ > *T*_*short*_) that includes both the prompt and slow components. We then tiled the light-output axis into energy slices and, in each slice, fit the PSP distribution with a Gaussian to obtain the centroid μ and width σ of the γ-band and, after excluding the γ-region, likewise for the neutron band. Smooth fiducial envelopes were generated by fitting these per-slice to low-order functions.

Neutrons were selected within a conservative band bounded below by the γ-band upper envelope and above by the neutron-band lower envelope. Operationally, the γ-rejection line was set at +3σ_γ_ and the neutron acceptance boundary at 5σ_n_, both evaluated as continuous functions of light output. This yields > 98% γ rejection with high neutron retention over the analysis range. Reaction yields for lower energies ( < 3.0 keV) were quantified in the proton channel only due to the much lower background rate in the Si diode detector compared to the EJ-309.

### Relative thick-target yield

For each center-of-mass energy, *E*_*cm*_, event totals were live-time corrected and background subtracted to obtain net counts *N*_*net*_*(E*_*cm*_*)*. Proton and neutron datasets were combined after channel-specific efficiency corrections, exploiting the equal D–D branching fractions (*p* + *t* and *n* + ^3^He) in this range. All results are expressed as relative yields, *R*_*exp*_*(E) = N*_*net*_*(E*_*cm*_*)/ N*_*net*_*(E*_*ref*_*)* with *E*_*ref*_ = 6.5 keV (in cm frame). Beam current was used to verify steady operating conditions. At low energies, the D–D fusion cross section is written as^1,26^:

7 $$\sigma \left(E,{U}_{e}\right)=\frac{S\left(E\right)}{\sqrt{E\left(E+{U}_{e}\right)}}\exp \left(-\sqrt{\frac{{E}_{G}}{\left(E+{U}_{e}\right)}}\right)$$

where *E* is the center-of-mass energy of colliding ions and *S(E)* is the astrophysical S-factor, a slowly varying function that describes the probability of a fusion event in the absence of Coulomb repulsion. The exponential term represents barrier penetrability with Gamow energy,

8 $${E}_{G}={2{m}_{r}{c}^{2}\left(\pi \alpha {Z}_{1}{Z}_{2}\right)}^{2}$$

where ${m}_{r}$ is the reduced mass of the reacting species, α the fine structure constant, and ${Z}_{1}$ and ${Z}_{2}$ the respective atomic numbers of fusing particles. The measured thick-target yield is the stopping-weighted integral

9 $$Y\left(E\right)={N}_{0}{\int }_{0}^{E}\sigma \left({E}^{{\prime} }\right){\left(-\frac{d{E}^{{\prime} }}{{dx}}\right)}^{-1}d{E}^{{\prime} }$$

with ${N}_{0}$ as the deuteron number density and -*dE/dx* the total stopping power in the membrane. Figure 2 reports the relative yield,

10 $${R}_{\exp }\left({E}_{{cm}}\right)=\frac{{Y}_{\exp }\left({E}_{{cm}}\right)}{{Y}_{\exp }\left({E}_{{ref}}\right)},\quad {E}_{{ref}}=6.5{{\rm{keV}}}\left({{\rm{cm}}}\right)$$

### Screening enhancement and model fit

Electron screening is quantified through the enhancement factor^15^

11 $${{\mathscr{E}}}\left({E}_{{cm}};{U}_{e}\right)\equiv \frac{{Y}_{{{\rm{scr}}}}\left({E}_{{cm}};{U}_{e}\right)}{{Y}_{{{\rm{bare}}}}\left({E}_{{cm}}\right)}$$

where ${E}_{{cm}}$ is the center-of-mass energy, ${Y}_{{{\rm{scr}}}}({E}_{{cm}};{U}_{e})$ is the measured fusion yield in the metallic target, and ${Y}_{{{\rm{bare}}}}({E}_{{cm}})$ is the yield expected for bare, unscreened nuclei^26^. By construction, ${{\mathscr{E}}}=1$ in the absence of screening.

For a homogeneous screening environment characterized by a single effective screening potential ${U}_{e},{\mbox{the}}\,{\mbox{enhancement}}\,{\mbox{takes}}\,{\mbox{the}}{\mbox{closed}}{\mbox{-}}{\mbox{form}}\,{\mbox{expression}}$

12 $${{{\mathscr{E}}}}_{{{\rm{scr}}}}\left({E}_{{cm}};{U}_{e}\right)=\sqrt{\frac{{E}_{{cm}}}{{E}_{{cm}}+{U}_{e}}}\exp \left[\sqrt{\frac{{E}_{G}}{{E}_{{cm}}}}-\sqrt{\frac{{E}_{G}}{{E}_{{cm}}+{U}_{e}}}\right]$$

Where ${E}_{G}$ is the Gamow energy for the reacting system. The screening potential ${U}_{e}$ acts as an effective upward shift of the reaction energy, increasing the Coulomb barrier penetrability. A single constant ${U}_{e}$, however, is insufficient to describe the full energy dependence of the measured yields as shown in Fig. 2. As the beam energy changes, ions sample different defect populations, deuterium concentrations, and local electronic environments as the implantation depth approaches the surface ($\sim 4{{\rm{nm}}}$ at ${E}_{{cm}}=0.25{{\rm{keV}}}$). The measured fusion yield at a given ${E}_{{cm}}$ therefore reflects a depth-weighted average over local reaction environments rather than a single uniform screening condition. The apparent screening strength extracted from the data should accordingly be regarded as an effective quantity that encodes the evolving mixture of local fusion environments sampled by the beam.

To represent this behavior while retaining a direct connection to screening physics, we model the measured enhancement as a weighted sum of contributions from two screened environments—denoted “surface” and “bulk”:

13 $${{{\mathscr{E}}}}_{{{\rm{env}}}}\left({E}_{{{\rm{cm}}}}\right)={A}_{s}{{{\mathscr{E}}}}_{{{\rm{scr}}}}\left({E}_{{{\rm{cm}}}};{U}_{e,s}\right)+{A}_{b}{{{\mathscr{E}}}}_{{{\rm{scr}}}}\left({E}_{{{\rm{cm}}}};{U}_{e,b}\right)$$

Here ${A}_{s},{A}_{b}\ge 0$ are dimensionless amplitudes for the surface and bulk channels, constrained to satisfy ${A}_{s}+{A}_{b}=1$, which ensure that ${{{\mathscr{E}}}}_{{{\rm{env}}}}\to 1$ in the absence of screening $\left({U}_{e,s}={U}_{e,b}=0\right)$. ${U}_{e,s}$ and ${U}_{e,b}$ are the effective screening potentials associated with the surface and bulk environments, respectively.

Using Eq. (12) for the homogeneous screening function, Eq. (13) may be written explicitly to yield the two-environment bi-exponential screening model,

14 $$ {{{\mathscr{E}}}}_{{{\rm{env}}}}\left({E}_{{cm}}\right) =	 {A}_{s}\sqrt{\frac{{E}_{{cm}}}{{E}_{{cm}}+{U}_{e,s}}}\exp \left[\sqrt{\frac{{E}_{G}}{{E}_{{cm}}}}-\sqrt{\frac{{E}_{G}}{{E}_{{cm}}+{U}_{e,s}}}\right] \\ 	+{A}_{b}\sqrt{\frac{{E}_{{cm}}}{{E}_{{cm}}+{U}_{e,b}}}\exp \left[\sqrt{\frac{{E}_{G}}{{E}_{{cm}}}}-\sqrt{\frac{{E}_{G}}{{E}_{{cm}}+{U}_{e,b}}}\right]$$

Within this formulation, the ${A}_{s}$ and ${A}_{b}$ are constant amplitudes, while the energy dependence of each channel’s contribution arises naturally through the ALR screening function—the Gamow factor causes the screened enhancement to increase sharply at low energies for larger ${U}_{e}$. To connect the bi-exponential model to a single screening parameter at each energy, we define an equivalent effective screening potential ${U}_{e,{eff}}({E}_{{cm}})$ as the unique homogeneous-screening value that would reproduce the same enhancement:

15 $${{{\mathscr{E}}}}_{{{\rm{scr}}}}\left({E}_{{cm}};{U}_{e,{eff}}\left({E}_{{cm}}\right)\right)={{{\mathscr{E}}}}_{{{\rm{env}}}}\left({E}_{{cm}}\right)$$

Equation (15) defines ${U}_{e,{eff}}({E}_{{cm}})$ implicitly—it is the unique screening potential that makes the homogeneous model reproduce the measured enhancement at each beam energy. To evaluate it explicitly, we parameterize ${U}_{e,{eff}}({E}_{{cm}})$ directly as a smooth sigmoid transition between the two screening environments of Eq. (13):

16 $${U}_{e,{eff}}\left({E}_{{cm}}\right)={U}_{e,b}+\frac{{U}_{e,s}-{U}_{e,b}}{1+\exp \left[-\left({E}_{{cm}}-{E}_{0}\right)/\Delta \right]}$$

where $\Delta=0.5{{\rm{keV}}}$ is a fixed smoothing parameter controlling the sharpness of the transition and ${E}_{0}$ (keV) is the crossover energy at which the two environments contribute equally to the measured yield. Substituting Eq. (16) into Eq. (12) gives the heterogeneous enhancement model evaluated at each beam energy. Fixing ${U}_{e,b}$ to the measured screening values reported in Fig. 2 anchors the high-energy behavior to a directly measured quantity, leaving only two free parameters that govern the low-energy departure.

The fit is performed in two stages. First, a global search over the parameter space is constructed using differential evolution to avoid local minima (population size 20, maximum 400 iterations, random seed 1234). The best-found solution is then refined using a trust-region lease-squares algorithm with soft −ℓ_1_ loss to reduce sensitivity to outliers. The resulting $${U}_{e,{eff}}\left({E}_{{cm}}\right)$$ profile directly encodes the physical picture: at high beam energies (deeper implantation depth), the sigmoid approaches $${U}_{e,b}$$, recovering the values uncovered in our experiments. At low beam energies (shallow implantation depths), it approaches $${U}_{e,s}$$, reflecting the stronger effective screening sampled by ions implanted in the near-surface, defect-rich region. The crossover energy ($${E}_{0}=0.6$$$${{\rm{keV}}}$$ in all cases) marks the transition between these two regimes and corresponds to the beam energy at which the surface and bulk screening environments contribute equally to the measured yield.

The model parameters $\theta=\left\{{A}_{s},{E}_{0,}{U}_{e,s}\right\}$, are determined by minimizing the weighted log-space residuals,

17 $${\chi }^{2}\left(\theta \right)={{\sum }_{i}\left[\frac{{{\mathrm{ln}}}({{\rm{R}}}_{i})-{{\mathrm{ln}}}({{{\mathscr{E}}}}_{{{\rm{env}}}}\left({{E}}_{{i}};\theta \right))}{{\sigma }_{{{\mathrm{ln}}}\,{{R}},{{i}}}}\right]}^{2}$$

where ${R}_{i}$ are the measured enhancement ratios at energies ${E}_{i}$ and ${\sigma }_{{{\mathrm{ln}}}\,{{R}},{{i}}}$ is the uncertainty in ${{\mathrm{ln}}}({R}_{i})$. Fitting in log-space ensures that residuals at low energies, where the enhancement spans many orders of magnitude, receive equal relative weight to those at high energies. For small fractional uncertainties,

18 $${\sigma }_{{\mathrm{ln}}R,i}\approx \frac{{\sigma }_{R,i}}{{R}_{i}}$$

with ${\sigma }_{R,i}$ the uncertainty in the measured enhancement ratio. The best-fit parameters shown in the supplementary table 2 are used to construct the model curves and the equivalent ${{\mathscr{E}}}\left({E}_{{cm}};{U}_{e}\right)$ and ${U}_{e,{eff}}\left({E}_{{cm}}\right)$ profiles shown in Fig. 3.

### Error propagation

Statistical counting uncertainties follow Poisson statistics with $\delta Y=\sqrt{N}/t$. For a ratio *R* = *Y/Y*_*ref*_, uncertainties propagate as:

19 $$\frac{\delta R}{R}=\sqrt{{\left(\frac{\delta Y}{Y}\right)}^{2}+{\left(\frac{\delta {Y}_{{{\rm{ref}}}}}{{Y}_{{{\rm{ref}}}}}\right)}^{2}}$$

Systematics common to all energies—solid angle, entrance-foil transmission, and stable electronics—cancel in both *R* and ${{\mathscr{E}}}$. Residual systematics, comprising beam-charge calibration stability, PSP selection boundaries, and diode energy-scale checks, are propagated into ${\sigma }_{R,i}$ and reflected in the confidence intervals on *U*_*e*_. Beam current uncertainties (± 2%) and loading stability (± 10 mV cathode potential) are treated as correlated across energies and propagated separately from the Poisson counting term, which dominates at low energies where absolute count rates are lowest. Residual systematic contributions entering through the energy dependence of stopping power are estimated to contribute less than 1% of the total uncertainty budget and are therefore neglected.

The enhancement factor ℰ(*E*) = *Y*_scr_(*E*)/*Y*_bare_(*E*) inherits the same relative yield uncertainties. The bare-nucleus reference *Y*_*bare*_ is computed from the Bosch–Hale parameterization^26^, which introduces no free parameters and contributes at the ~1% level. The ±0.2 keV uncertainty on *U*_*e*_ represents a combined statistical and systematic estimate propagated through the full fitting procedure.

### Post-exposure structural analysis

X-ray diffraction was performed ex situ on a Rigaku SmartLab (Cu K_α_, λ = 1.5406 Å) using identical geometry before and after irradiation. For Pd, the diffractograms from pristine and exposed regions are indistinguishable within instrumental resolution: the face-centered-cubic (FCC) reflections [(111), (200), (220), (311), (222)] are conserved in position and relative intensity. We therefore conclude that Pd retains its FCC lattice during the experiments and that any beam-induced modifications are limited to defect/strain fields rather than a change in crystalline phase.

For Ti, pristine areas display the expected α-Ti hexagonal-close-packed (HCP) pattern [(100), (002), (101), (102), (110), (103), (112), (201), (004)]. After exposure, irradiated regions exhibit additional reflections that index to δ-TiD_x_ (FCC hydride), while α-Ti peaks persist with reduced prominence. The appearance of δ-TiD_x_ is consistent with substantial deuterium uptake under our combined ion beam implantation and electrochemical loading; at room temperature, the δ-field corresponds to x ≈ 1.6−1.9 (D/Ti)^55^. In both Pd and Ti, post-exposure line widths increase modestly, consistent with elevated microstrain and reduced coherent domain sizes near the surface.

Collectively, the XRD results support two methodological conclusions. First, the Pd membrane retains its FCC phase across runs, removing phase evolution as a confound in yield comparisons. Given Pd’s high hydrogen diffusivity at room-temperature, deuterium introduced during operation readily redistributes and deloads. Therefore, post-run structural changes are dominated by near-surface defect/strain rather than stable hydride formation. Second, Ti develops a δ-TiD_x_ hydride layer confined to the near surface, consistent with the elevated deuterium chemical potential in our protocol and with Ti’s substantially lower hydrogen diffusivity; deuterium therefore resides longer in the Ti lattice, favoring hydride stabilization and providing a clear structural context for the observed changes in low-energy transport and screening in the Ti system.

## Supplementary information

Supplementary Information

Transparent Peer Review file

## Data Availability

The data generated in this study are available in the Zenodo database under accession code 10.5281/zenodo.20435774
