# Supplementary material for: Enhanced nuclear fusion in the sub-keV energy regime
Source: Nat Commun. 2026 Jul 18;17:8845. doi: 10.1038/s41467-026-74421-1 (PMC13500673; doi:10.1038/s41467-026-74421-1)
Supplement: Supplementary file 1 [file 41467_2026_74421_MOESM1_ESM.pdf]

Supplementary Information:
Enhanced nuclear fusion in the sub-keV energy regime

Micah E. Karahadian[1], Matthew Colborne[2], Arun Persaud[2], Thomas Schenkel[2], Jeremy N. Munday[1]

[1]Department of Electrical and Computer Engineering, University of California, Davis, One Shields Avenue, Davis, CA 95616-5270, USA.
[2]Accelerator Technology and Applied Physics Division, Lawrence Berkeley National Laboratory, 1 Cyclotron Road, Berkeley, CA 94720, USA.

Supplementary Figures

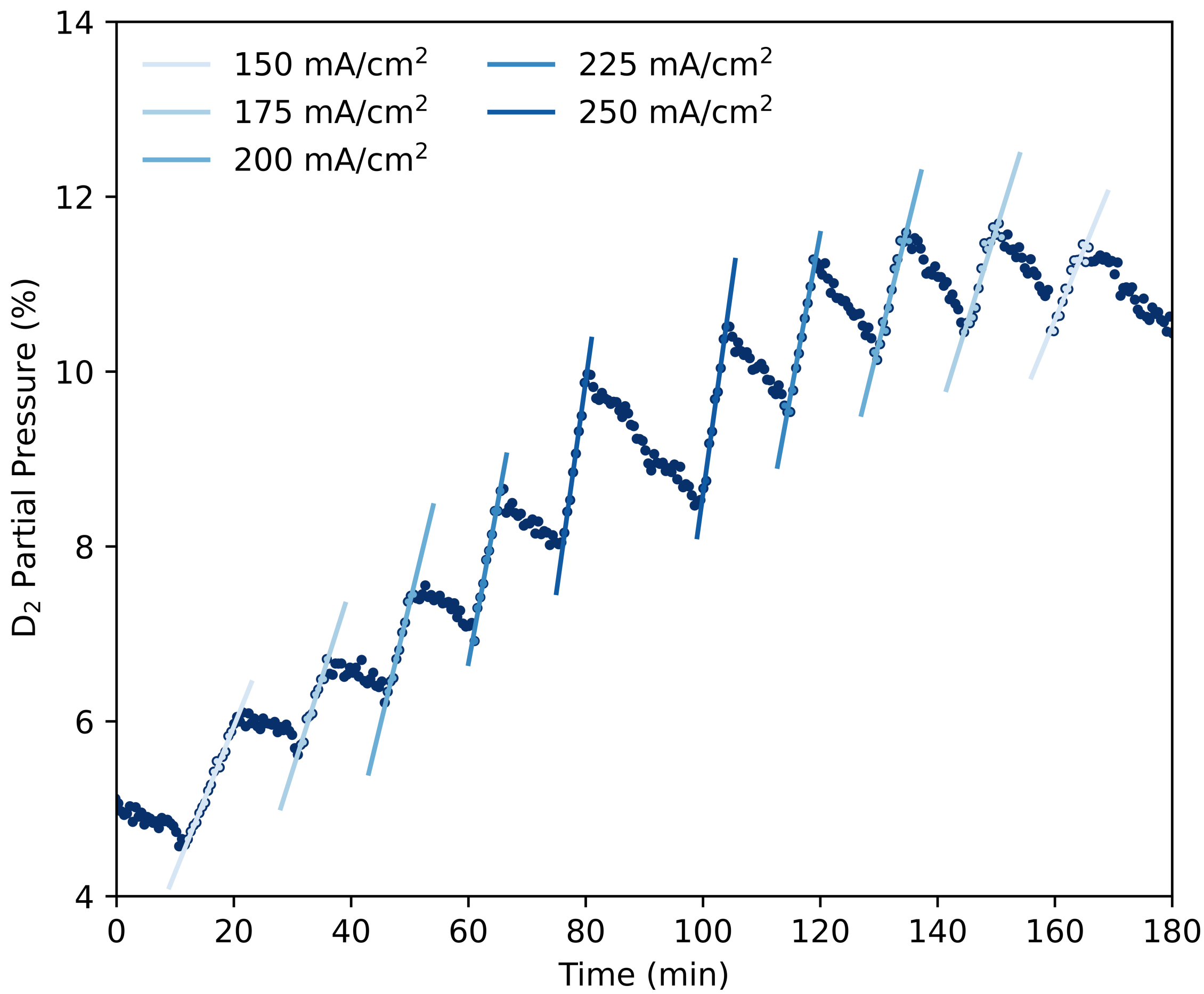

Supplemental Figure 1 | Linear dependence of D_2 flux with applied current density. Residual-gas analysis (RGA) trace of the D_2 (4 amu) partial pressure during a stepped galvanostatic ramp in 6M D_2SO_4 across a 250 µm Pd membrane. Each light-blue segment marks a constant-current step (150-250 mA cm^{-2}); the pressure increases linearly within each step, and the slope scales with the applied current density, indicating a current-controlled D_2 outflux into vacuum, confirming stable, current-controlled deuterium delivery to the membrane.

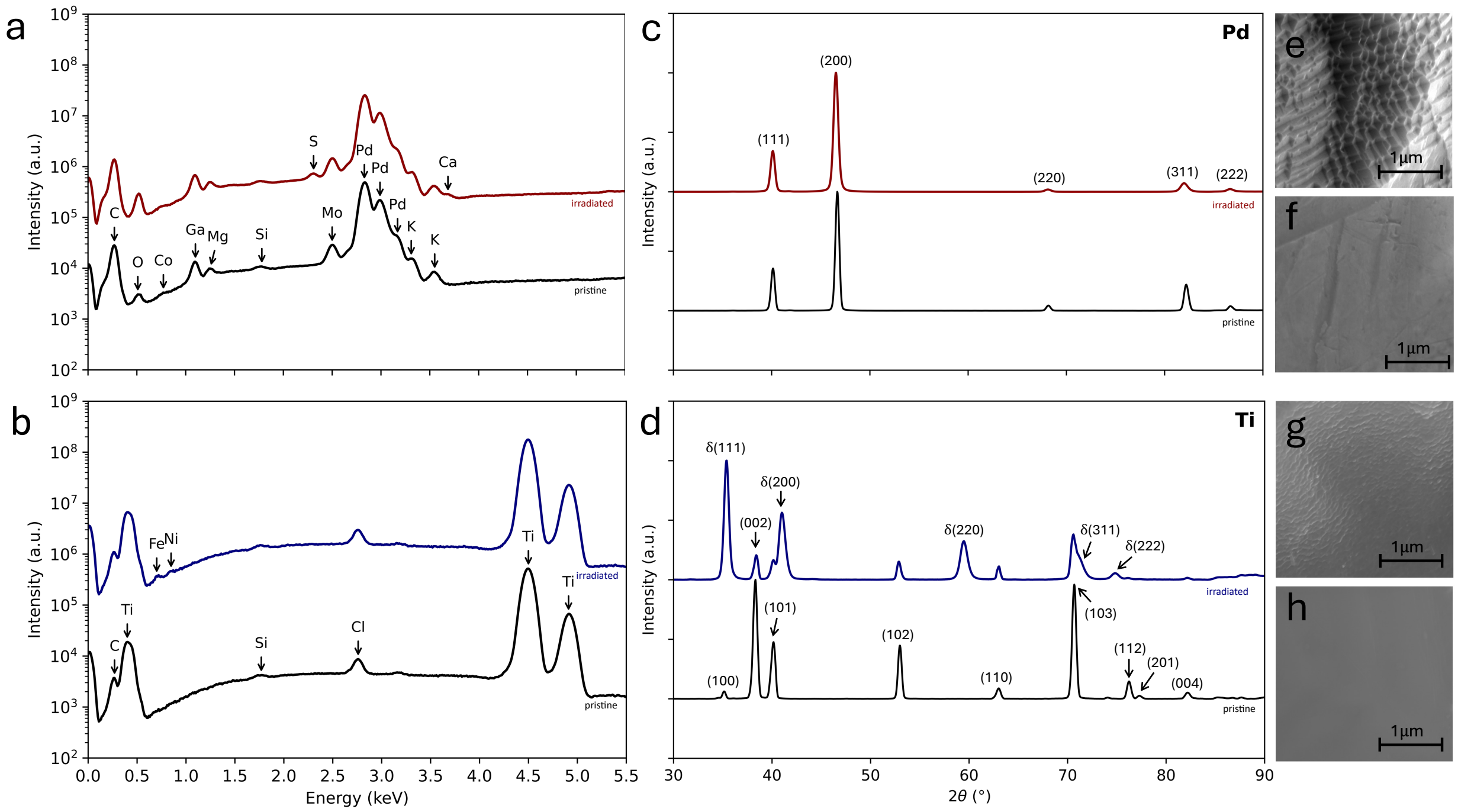

Supplemental Figure 2 | Surface composition and lattice signatures before and after beam exposure. (a, b) Energy-dispersive X-ray spectra (log intensity) from palladium (a) and titanium (b) foils, comparing pristine (black) and bombarded regions (Pd: red; Ti: blue). Major metal lines are unchanged in position and relative intensity after bombardment. Low-energy variations across both samples are consistent with typical surface adsorbates. Beyond trace amounts of S and Ca in the Pd case (electrolyte) and Fe/Ni in the Ti case (chamber sputtering), no new heavy elements were detected, indicating the bulk composition remains stable after bombardment within detector resolution. **(c, d)** X-ray diffraction from the same foils. For Pd (c), both scans exhibit the face-centered-cubic (FCC) reflections (111), (200), (220), (311), and (222); peak positions are conserved, indicating the Pd lattice remains FCC after irradiation. For Ti (d), the pristine foil shows α-Ti hexagonal-close-packed (HCP) peaks [(100), (002), (101), (102), (110), (103), (112), (201), (004)], whereas the bombarded region exhibits additional FCC hydride reflections δ-TiD_x [(111), (200), (220), (311), (222)], consistent with residual hydride formation near the surface while α-Ti peaks persist from the underlying bulk. **(e-h)** SEM of representative areas showing increased surface texture after bombardment for beam exposed Pd (e) and pristine (f) as well as beam-exposed Ti (g) and the pristine counterpart (h).

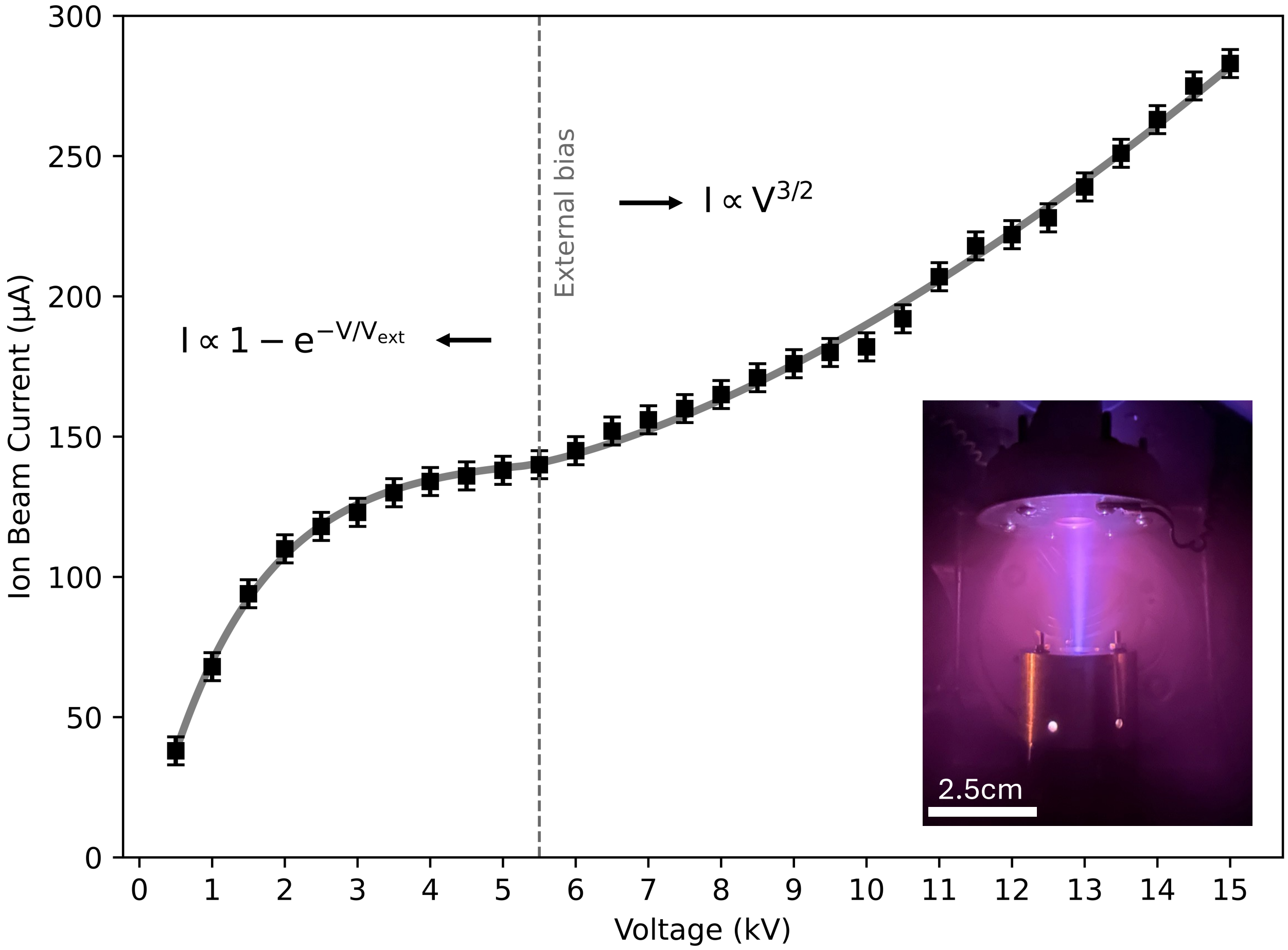

Supplemental Figure 3 | Ion-beam I–V characteristic and operating regimes. Beam current was recorded with a Faraday cup at the target plane while the three-electrode extraction optics were held at constant extraction voltage. A transition is observed above 5.5 kV (vertical dashed line), the onset of external target bias. For $V \leq 5.0$ kV the beam is transported under internal gun bias only, and the current follows a saturating exponential $I \propto 1 - \exp(-V/V_{ext})$, consistent with fixed extraction optics. For $V \geq 5.5$ kV, increasingly negative target bias produces electrostatic focusing that narrows the beam at the cup entrance; with a fixed-diameter cup this increases the collected fraction and yields the observed $I \propto V^{3/2}$ dependence, demonstrating that beam transport and energy delivery remain well controlled across the sub-keV to keV regime. Error bars represent the spread of repeated measurements (including readout noise). Inset: photograph of the dual-chamber apparatus showing the electrochemical cell (top) and ion source (bottom).

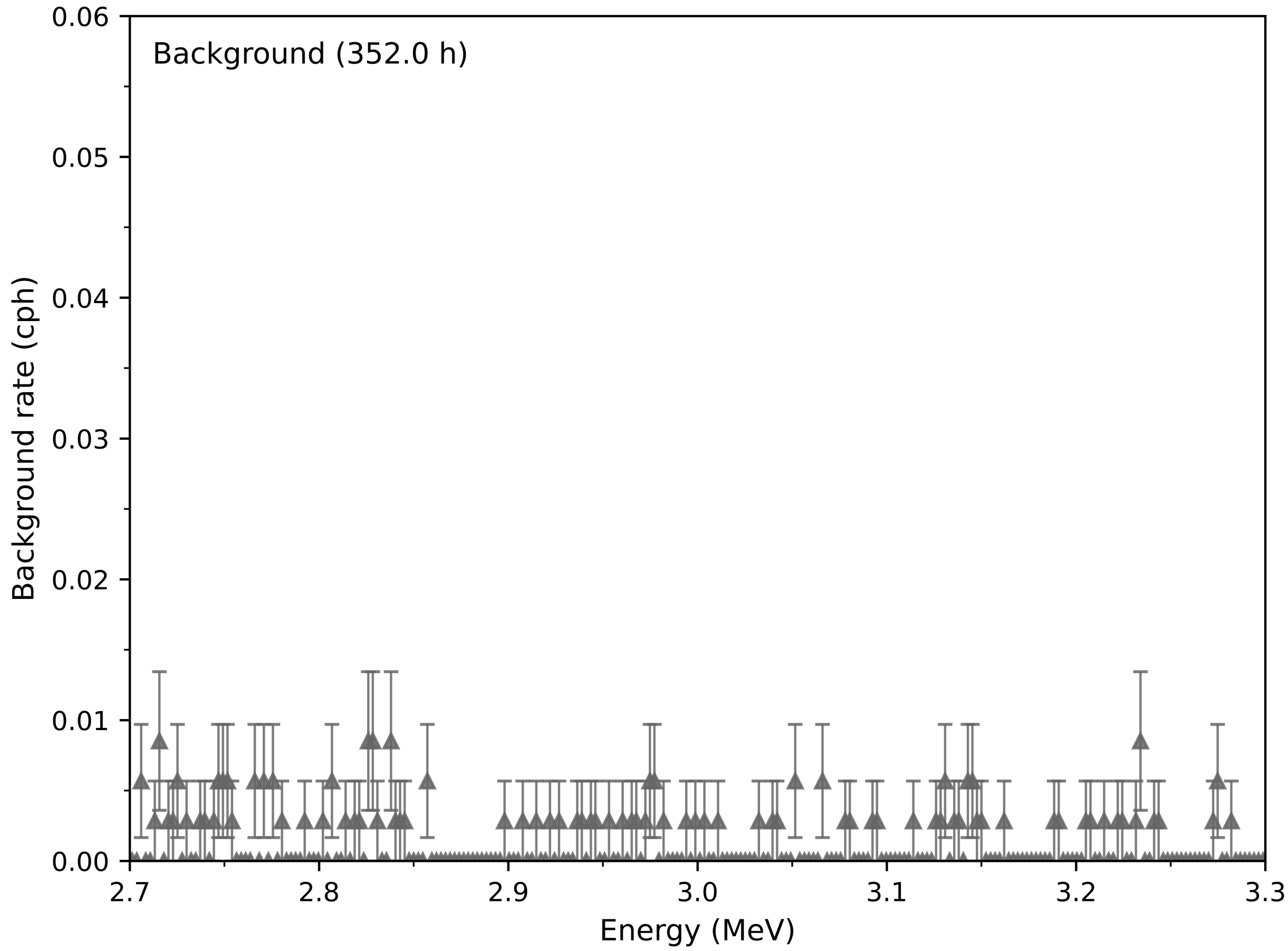

Supplemental Figure 4 | Background proton energy spectrum. Background accumulated over 352 hours of beam-off, electrochemistry-off operation. The background rate is stable at ~0.007 counts per hour (cph) across the full proton integration window (2.7–3.3 MeV), with no anomalous structure near the 3.02 MeV fusion proton peak. Error bars denote 1σ Poisson counting uncertainties.

Supplementary Tables

E_{cm} (keV)	I (μA)	Pd (S:B)	PdD (S:B)	Ti (S:B)	TiD (S:B)
**3.0**	145	443:1	465:1	1525:1	5427:1
**2.5**	138	121:1	177:1	225:1	1082:1
**2.0†**	134	83:1	138:1	82:1	368:1
**1.5†**	123	53:1	92:1	106:1	261:1
**1.0†**	110	44:1	43:1	69:1	112:1
**0.5†**	68	15:1	32:1	47:1	62:1
**0.25†**	38	21:1	30:1	15:1	56:1

Supplementary Table 1 | Background comparison. Signal-to-background ratios (S:B) for each beam energy and material condition, computed as the signal rate in counts per hour (cph) divided by the background rate (~0.007 cph) in the proton integration window (2.7–3.3 MeV). Signal rates are derived from measured yields (counts/Coulomb) and beam currents from the ion source IV characterization. †Rows denote the plateau region ($E_{cm} \leq 2$ keV) where the yield ceases to fall exponentially with energy.

Material	$U_{e,s}$ (keV)	$U_{e,b}$ (keV)	E_0 (keV)
Pd	3.28	0.8	0.6
PdD	3.68	1.7	0.6
Ti	3.21	0.3	0.6
TiD	2.75	0.9	0.6

Supplementary Table 2 | Best-fit heterogeneous screening model parameters. Best-fit parameters of the sigmoid screening model (Eq. 16) for each material condition. $U_{e,s}$ is the low-energy (surface) asymptotic screening potential, $U_{e,b}$ is the high-energy (bulk) asymptotic screening potential fixed to the measured value for each material, and E_0 is the crossover energy at which the surface and bulk screening environments contribute equally to the measured yield.
