# Supplementary material for: Enhanced nuclear fusion in the sub-keV energy regime
Source: Nat Commun. 2026 Jul 18;17:8845. doi: 10.1038/s41467-026-74421-1 (PMC13500673; doi:10.1038/s41467-026-74421-1)
Supplement: Supplementary file 2 [file 41467_2026_74421_MOESM2_ESM.pdf]

nature portfolio

# Peer Review File

# Enhanced nuclear fusion in the sub-keV energy regime

Corresponding Author: Professor Jeremy Munday

This manuscript has been previously reviewed at another journal. This document only contains information relating to versions considered at Nature Communications. Mentions of the other journal have been redacted.

Version 0:

Reviewer comments:

Reviewer #1

(Remarks to the Author)
The authors have satisfactorily addressed most of the reviewers' comments on the previous manuscript submitted to [*REDACTED*]. The modified manuscript is suitable for publication in Nature Communications.

Reviewer #2

(Remarks to the Author)
The revised manuscript reports measurements of deuterium–deuterium fusion in metallic hydrides at sub-keV energies. It presents evidence for a pronounced low-energy enhancement relative to standard bare-nucleus expectations. The reported plateau-like behaviour below ~2 keV is experimentally interesting and potentially relevant for the broader study of low-energy nuclear reactions in condensed-matter environments.

Overall, the revised manuscript satisfactorily addresses the major concerns raised during the previous review process. In particular, the authors have substantially improved the discussion of the physical interpretation of the observed plateau-like enhancement, clarified the treatment of electron screening and the modelling and fitting procedures, and provided significantly expanded methodological detail regarding background subtraction. The additions to the Methods and Discussion sections have improved both the transparency and reproducibility of the analysis. The authors also appropriately acknowledge the limitations of the current work.

In my opinion, the methodology is generally sound and the experimental procedures are described in sufficient detail for the work to be reproducible within the standards of the field. While the underlying physical mechanism responsible for the observed plateau-like behavior below ~2 keV remains an open question, I believe the manuscript will be of interest to researchers across several areas of physics, including low-energy nuclear physics, condensed-matter physics, and fusion-related studies, and that it is suitable for publication in Nature Communications.

Reviewer #3

(Remarks to the Author)

Reviewer #4

(Remarks to the Author)
The changes made by the authors to the manuscript originally submitted to [*REDACTED*] are satisfactory. In my opinion, the new version of the manuscript is suitable for publication in Nature Communications.

**Open Access** This Peer Review File is licensed under a Creative Commons Attribution 4.0 International License, which permits use, sharing, adaptation, distribution and reproduction in any medium or format, as long as you give appropriate credit to the original author(s) and the source, provide a link to the Creative Commons license, and indicate if changes were made.
In cases where reviewers are anonymous, credit should be given to 'Anonymous Referee' and the source.
The images or other third party material in this Peer Review File are included in the article's Creative Commons license, unless indicated otherwise in a credit line to the material. If material is not included in the article's Creative Commons license and your intended use is not permitted by statutory regulation or exceeds the permitted use, you will need to obtain permission directly from the copyright holder.
To view a copy of this license, visit https://creativecommons.org/licenses/by/4.0/
